# Prevalence of coronary artery disease risk factors in Iran: a population based survey

**DOI:** 10.1186/1471-2261-7-32

**Published:** 2007-10-30

**Authors:** ZN Hatmi, S Tahvildari, A Gafarzadeh Motlag, A Sabouri Kashani

**Affiliations:** 1 Medical faculty, Tehran University/medical sciences, Tehran, Iran; 2 Institute of public health Tehran university/public health, Tehran, Iran; 3 Medical faculty, Tehran University/medical sciences, Tehran, Iran; 4 Curriculum Unit in EDC, Tehran university of medical sciences, Tehran, Iran

## Abstract

**Background:**

Coronary artery disease (CAD) is a leading cause of mortality, morbidity, and disability with high health care cost in Iran. It accounts for nearly 50 percent of all deaths per year. Yet little is known about CAD and CAD risk factors in the Iranian population. We aimed to assess the prevalence of different CAD risk factors in an Iranian population.

**Methods:**

A descriptive cross sectional survey was conducted involving 3000 healthy adults at 18 years of age or above who were recruited with cluster random sampling. Demographic data and risk factors were determined by taking history, physical examination and laboratory tests.

**Results:**

The average age was 36.23 ± 15.26. There was 1381 female (46%) and 1619 male (54%) out of which 6.3% were diabetic, 21.6% were smoker, and 15% had positive familial heart disease history. 61% had total cholesterol level > 200 mg/dL, 32% triglyceride > 200 mg/dl, 47.5% LDL-c > 130 mg/dl, 5.4% HDL-c < 35 mg/dl, 13.7% systolic blood pressure > 140 mmHg, 9.1% diastolic blood pressure > 90 mmHg and 87% of them were physically inactive.

**Conclusion:**

Clinical and Para-clinical data indicated that Iranian adult population are of a high level of CAD risk factors, which may require urgent decision making to address national control measures regarding CAD.

## Background

CAD is a leading cause of mortality, morbidity, and disability in Iranian population. It accounts for nearly 50 percent of all deaths per year. CAD is characterized by the presence of atherosclerosis in the epicardial coronary arteries. Atherosclerotic plaques, the hallmark of atherosclerosis, progressively narrow the coronary artery lumen and impair ante grade myocardial blood flow. The reduction in coronary artery flow may be symptomatic or asymptomatic, may occur with exertion or at rest, and may culminate in a myocardial infarction, depending on obstruction severity and the rapidity of its development [1]. CAD is the most common form of cardiovascular disease with an estimated prevalence of CAD in men is 6.9% and 6% among women [1]. For people at 18 years of age and above prevalence estimates are: only 11.4 percent among whites. While 5.9 percents suffered from heart disease, 5.9 percent have hypertension and 2.3 percent have had a stroke [2].

Among African Americans black only, 9.9 percents had heart disease, 5.3 percents CHD,31.6 percents had hypertension and 3.5 percents had experienced a stroke [2]. Among Hispanics or Latinos, 7.7 percents had heart disease, 4.5 percents have CHD, 19.0 percents had hypertension and 2.2 percents had a stroke[2]. Among Asians, 5.6 percents had heart disease, 3.8 percents had CHD, 16.1 percents had hypertension and 1.8 percents a stroke[2]. In South Asia the prevalence of hypertension is 3.2 percent, diabetes 2.6 percent, and CAD is 3.2 percent. However, in Urban and immigrant populations the prevalence rates are, 12–20 percents, 6–8 percents and, 7–14 percents respectively. Mean serum cholesterol level is 180–200 mg/dl, frequency of obesity 5–8% and dietary fat intake contains 20–30% of total calorie intake[3]. Several subgroups of South Asia do have high smoking rates, especially in Urban areas. The prevalence of cigarette smoking in Indians was 1.3% as compared with 27% in whites in the UK, cholesterol levels among Indians were found to be lower than the native population, while in the USA total cholesterol and LDL-c level among Indians and whites were similar. In a study of migrant Indians physicians to the USA, mean level of HDL-c level were significantly less in younger Indian men and women than their Western counterparts. Similar trends have been found for triglycerides as well[4]. Among the known risk factors, levels of HDL-c have been found to be inherently low in the normal South Asians population. Low HDL-c alone or in combination with high levels of LDL-c, insulin resistance and hypertension constitute a very important intermediate phenotype for CAD. In a recent study on young myocardial infarction patients, 70.3% were found to have HDL-c lower than 40 mg/dl [5].

The average annual rates for the first major cardiovascular events rise from seven per 1000 men at 35 – 44 years of age to 68 per 1000 at 85 – 94 years of age. For women, comparable rates occur 10 years later in life[6]. Preliminary mortality data proved CVD as the underlying cause of death accounting for 37.3 percent of all deaths, or one of every 2.7, in the United States in 2003. CVD as an underlying or contributing cause of death accounted for about 58 percent of the deaths in 2002 [1]. Data from the 2003 BRFSS study of adults of 18 years of age and above showed the prevalence of respondents reporting two or more risk factors for heart disease and stroke increased among successive age groups. The prevalence of having two or more risk factors was highest among the blacks (48.7 percents) and American Indians/Alaska Natives (46.7 percents) and lowest among Asians (25.9 percents), the prevalence was similar in women (36.4 percents) and men (37.8 percents) were similar[1]. The estimated direct and indirect costs for coronary heart disease in 2006 is $142.5 billion[1].

CAD is a chronic process that begins during adolescence and slowly progresses throughout life. Independent risk factors include a family history of premature CAD, cigarette smoking, diabetes mellitus, hypertension, dyslipidemia, a sedentary life style, advanced age, gender and obesity. The risk factors accelerate or modify a complex and chronic inflammatory process that ultimately manifests as fibrous atherosclerotic plaque [1]. The Incidence of CAD is compatible with the pattern of the distribution of CAD risk factors, CAD occurs when its risk factors are present. According to a case-control study of 52 countries (INTER HEART), nine easily measured and potentially modifiable risk factors accounts for over 90 percent of the risk of an initial acute myocardial infarction (MI). The effect of these risk factors is consistent in men and women, across different geographic regions, and by ethnic group, making the study applicable worldwide. These nine risk factors include cigarette smoking, abnormal blood lipid levels, hypertension, diabetes, abdominal obesity, a lack of physical activity, low daily fruit and vegetable consumption, alcohol over consumption, and the psychosocial index[7].

This study utilized a population based survey to measure the prevalence of different CAD risk factors in an Iranian population sample where little is known about CAD, the disease burden, and risk factors.

## Methods

A cross sectional survey was conducted to assess the prevalence of different CAD risk factors utilizing the medical history, physical examination and laboratory tests to consider known risk factors. In a population based survey among the general population in Tehran, Iran we applied cluster random sampling and calculated 3000 for the sample population. We collected the data regarding risk factors for all healthy individuals (regarding to the public health school on London questionnaire) of 18 years of age and above. After completing the informed consent (verbal consent), all participants were given an interview, physical examination and blood sample tests. Per protocol blood samples were obtained for FBS, total cholesterol, LDL-c, HDL-c, and triglyceride(TG). FBS were assayed at the enzymatic methodology by glucose oxidize Kid. Total cholesterol and TG assayed with enzymatic methodology and HDL-c level was measured by poly cat++ precipitating methodology. Study protocol was approved by ethical committee of the medical faculty.

### Statistical analysis

Descriptive analysis was used to process the outcomes in tables and graphs. Frequency tables was used to describe the categorical variables, and calculation of the means and SD was performed to describe the continuous variables

## Results

The 3000 persons with a mean age and standard deviation of 36.23 ± (15.26) respectively were analyzed out of which 46 percent (1381) were female and 54 percent (1619) were male.

Table 1 demonstrate the prevalence of risk factors under the study including, diabetes, total cholesterol, triglyceride, LDL-c, HDL-c, systolic and diastolic blood pressures, family history of CAD, cigarette smoking and physical inactivity.

**Table 1:** Prevalence of CAD risk factors

Risk factors	Frequency	Total
Diabetes	6.3% (189)	3000
Total cholesterol > 200 mg/dl	61% (1830)	3000
Triglyceride > 200 mg/dl	32% (960)	3000
LDLC > 130 mg/dl	45.5% (1365)	3000
HDLC < 35 mg/dl	5.4% (162)	3000
Systolic blood pressure > 140 mmHg	13.7% (411)	3000
Diastolic blood pressure > 90 mmHg	9.1% (273)	3000
Family history of CAD in first degree relatives	15% (450)	3000
Cigarette smoking (active smoker)	21/6% (648)	3000
Sedentary life style	87% (2610)	3000

LDL-c: Low Density Lipoprotein

HDL-c: High Density Lipoprotein

C.A.D : Coronary Artery Disease

mg/dl : Milligram per Deciliter

Table 2 presents the description of number, mean and standard deviation of the quantity of the risk factors.

**Table 2:** Distribution of some risk factors

Risk factors	Total	Mean	SD
FBS (mg/dL)	3000	113.98	± 37.02
Total cholesterol (mg/dL)	3000	220.64	± 55.34
Triglyceride (mg/dl)	3000	196.83	± 92.29
HDLC (mg/dl)	3000	41.68	± 13.24
LDLC (mg/dl)	3000	128.15	± 41.57

F.B.S : Fasting Blood Sugar

LDL-c: Low Density Lipoprotein

HDL-c: High Density Lipoprotein

mg/dl : Milligram per Deciliter

Table 3 shows the prevalence of diabetes, cigarette smoking (CS) and physical inactivity, blood pressure and dyslipedemia for the two genders.

**Table 3:** Prevalence of risk factors in female and male

Gender/ Risk factors	Diabetes	CS	Physical Inactivity	Systolic BP > 140 mmHg	Dyslipedemia
Female	3.21% (97)	2.5% (85)	48% (1440)	7.8% (234)	34% (1020)
Male	2.09% (93)	18.9% (599)	39% (1170)	5.9% (177)	26% (780)
Total	6.3% (190)	21.6% (684)	87% (2610)	13.7% (411)	60% (1800)

CS: Cigarette Smoking

BP : Blood Pressure

mmHg : Millimeter Hg

Diabetes was more common within the age group of 46–55 years, and in individuals who were inactive (exercise less than 30 minutes, three times a week) in comparison with physically active persons. (82.1% versus 17.9%)

Prevalence of hypertension (blood pressure ≥ 140/90 mmHg) was higher in female than in male, and within 56–65 age group of our sample. Of those who had a sedentary life style 82.2% suffered from hypertension among whom 17.8 percents did exercise.

In this study, physical inactivity was found to be more common among the females, and the illiterate or persons with an educational level lower than high school compared with high schoolers and those with a university degree, (37% versus 63%)

There were 9.9 percents of women who had a history of oral contraceptive pills (OCP) use and .34.2 percents of whom had a history of more than ten years of OCP use.

Females more than males were dislipedemic, and the prevalence of physical inactivity was higher in patients with dyslipidemia in comparison with those of a normal level of lipids (75.3% versus 24.7%).

## Discussion

CAD is the epidemic of our time and set to remain the single most important disease in the world in the terms of mortality, morbidity, disability and economic loss until 2020 year [8]. This chronic disease has an enormous impact on quality of life. Its Risk factors accelerate or modify a complex and chronic inflammatory process that ultimately manifest as fibrous atherosclerotic plaque [1], the clinical coronary events follow plaque rupturing. The presence and the number of CAD risk factors predict the future cardiovascular events in individuals with such factors.

In the present study we conducted a population based survey to assess the prevalence of CAD risk factors, which is the only study of this nature in Iran, and the results of the study revealed the high frequency of risk factors in our sample.

The development of the concept of risk factors and their relationship to the incidence of CAD evolved from prospective epidemiological studies in the United States and Europe [9-12]. The identification of risk factors provides a means for decreasing CAD risks, through the reduction of modifiable risk factors, and better treatment decisions, through more accurate determination of overall risk status [13]. Risk factors reduction is the primary clinical approach to preventing CAD morbidity and mortality[14]. Epidemiological studies have clearly demonstrated that, hypertension, use of tobacco and dyslipidemia are the major risk factors of CAD which act in a synergistic manner[14]. With the prevalence of risk factors for CAD is increasing, and with the clinical and cost burdens mounting, identifying and treating those at risk remains a national priority[15,16].

Comparison of the present study with KANALA in 2002[17] and former research in US and Europe [7,9,10,14] revealed a similar pattern of the prevalence of a number of risk factors, so that our population have a much higher level in such risk factors as, total cholesterol, LDL-c and physical inactivity. Also in hypertriglyceridemia and low HDL-c frequency, we find out a different pattern [17].

A comparison between our study and that of heart disease and stroke statistics 2006 up date[1], indicate that the prevalence of hypercholestrolemia, LDL-c equal than 130 mg/dl or higher and physical inactivity is significantly higher in our population. This huge difference might be addressed in the epidemic aspects of CAD arguments[1]. Our results and the above report[1]demonstrate a similar prevalence of diabetes and cigarette smoking. Low level of HDL-c level is much more common in the 2006 report of heart disease and stroke statistics, than that in our population[1].

A high prevalence of CAD risk factors, as expected, exist which is in line with economic development. The presence of a high level of most risk factors along with the absence of national and local area control measures in our population, strongly predicts the epidemic status of CAD in this population in the next future. The results of this study may lead to a decision made to address the issue at a national level. Control programs regarding CAD, via modifying the modifiable risk factors are envisaged.

According to the current ATP III guidelines, determining fasting lipid levels together with the presence of clinical signs and other known risk factors would generate an estimated 10 year risk for CAD. On the basis of this risk assessment, aggressive life style and treatment guidelines are recommended [15].

Recent scientific evidence [18-22] together with the joint AHA/CDC guidelines [15], however, strongly recommends that in performing a global risk assessment for CAD, new risk factors to measure heart attack risk such as circulating markers of inflammation like C-reactive protein (CRP)should be used. Such augmentation provides the best method of determining the global risk assessment for CAD.

For primary prevention purposes individuals with documented risk factors should actively pursue risk factor modifications to reduce the risk of future cardiovascular events. Life style modifications with guidance and strong encouragement of the physician-led health care team, can make a significant positive impact on the future cardiovascular and vascular event rates.

## Conclusion

According to the prevalence of risk factors, which 6.3% of the study population were diabetic, 21.6% were smoker, and 15% had a positive familial heart disease.61% had total cholesterol level > 200 mg/dl, 32% triglyceride > 200 mg/dl, 47.5% LDL-c > 130 mg/dl, 5.4% HDL-c < 35 mg/dl, 13.7% systolic blood pressure > 140 mmHg, 9.1% diastolic blood pressure > 90 mmHg and 87% of them were physically inactive. The researchers strongly recommend whole population strategy of primary prevention of the cad to be designed and practiced. Managing this burden of risk factors can prevent the following cardiovascular event rates, and should assist managing the future CAD.

## Competing interests

The author(s) declare that they have no competing interests.

## Authors' contributions

ZNH proposed the idea, designed the study, analyzed the data, prepared the manuscript and monitored the whole process from designing to preparing the manuscript. ST applied the health questionnaire and collection of the data. AG carried out the data collection, history taking and clinical examination, AS edited the prepared manuscript. Lab Staff were remunerated for the LAB tests. All authors read and approved the final manuscript.

## Pre-publication history

The pre-publication history for this paper can be accessed here:


